# Impact of diabetes mellitus on epithelial ovarian cancer survival

**DOI:** 10.1186/s12885-018-5162-3

**Published:** 2018-12-12

**Authors:** Setareh Akhavan, Akram Ghahghaei-Nezamabadi, Mitra Modaresgilani, Azam Sadat Mousavi, Mahdi Sepidarkish, Afsaneh Tehranian, Elahe Rezayof

**Affiliations:** 1 Gynecology Oncology Department, Vali-Asr Hospital, Tehran University of Medical Sciences, P.O. Box: 16635148, Tehran, Iran; 2 Department of Epidemiology and Reproductive Health, Reproductive Epidemiology Research Center, Royan Institute for Reproductive Biomedicine, ACECR, Tehran, Iran; 3 Department of Obstetrics and Gynecology, Roointan-Arash Women’s Hospital, Tehran University of Medical Sciences, Tehran, Iran; 4 Reproductive Health Research Center, Tehran University of Medical Sciences, Tehran, Iran

**Keywords:** Diabetes mellitus, Epithelial ovarian cancer, Overall survival, Progression free survival

## Abstract

**Background:**

Diabetes mellitus (DM) is associated with poorer outcomes in some cancers. Its effect on ovarian cancer is less clear. We consider the effect of DM on overall survival (OS) and progression free survival (PFS) in patients with epithelial ovarian cancer (EOC).

**Methods:**

A retrospective cohort study of 215 patients with EOC diagnosed between 2009 and 2016 was performed. Records were reviewed for standard demographic, pathologic and DM diagnosis data. Cox regression was used to evaluate the relationship between disease status and survival after adjustment for age, body mass index (BMI), parity, stage, grade, histology, debulking status, hypertension (HTN), menopause status and neoadjuant chemotherapy.

**Results:**

Patients with DM (27.97, 95%CI: 23.63 to 32.30) had a significantly shorter OS rates compared to patients without DM (41.01, 95%CI: 38.84 to 43.17). The unadjusted hazard ratio (HR) for the association between OS time and DM was 4.76 (95%CI: 2.99 to 7.59, *P* < 0.001). Following adjustment for demographic and pathologic variables, the HR was 3.93 (95% CI: 2.01 to 7.68; *P* < 0.001). The PFS in patients with DM (14.10, 95%CI: 11.76 to 16.44) was significantly shorter compared to patients without DM (28.83, 95%CI: 26.13 to 31.54). The unadjusted HR for PFS and DM was 5.69 (95% CI: 3.05 to 10.61; *P* < 0.001). After adjustment for demographic and pathologic variables, the HR was 2.73 (95% CI, 1.18 to 6.95; *P* < 0.001).

**Conclusions:**

DM can negatively effect on PFS and OS in EOC patients independent of the effect of other variables.

## Background

DM is increasing in the global population and also in ovarian cancer patients. Nowadays, approximately 9% of adults are living with diabetes worldwide. It has been estimated that the prevalence of DM would increase gradually, and the number of DM patients will exceed 600 million people by 2040 [1]. Many studies proposed that diabetes will increase the risk of many cancers and can decrease the cancer patients’ survival [2]. Ovarian cancer is the sixth most common cancer in the woman and has the highest mortality rate among all gynecological cancer [3, 4]. Every year over 100,000 ovarian cancer patients die of diabetes worldwide [5]. The incidence of ovarian cancer is increasing by age and is approximately five times higher in women over 65 years of ages [6]. Recent research demonstrated that 5-year survival rate of ovarian cancer was lower than 50%. Diabetes mellitus may be apart from the invasion of tumor cells and lead to this unfavorable prognosis [7]. More recently, medical conditions such as diabetes are considered, in addition to prognostic factors of tumor characteristics (histology and grade), patient’s characteristics (age and performance states), and treatment variables (surgical cytoreduction and chemotherapy) to evaluate the impact on both the incidence and outcome in patients with EOC [1, 8–11]. From the molecular aspect, data suggest that increased insulin growth factor-1 (IGF-1) raises cytokine and estrogen levels, adipokinase imbalance, and hyper insulinemia; then increase risk of malignancy and has an influence on survival in cancer [12–17]. Interestingly, metformin, the first line medication for the treatment of type 2 diabetes, particularly in overweight patients, [18, 19] has positive impact on survival in diabetic ovarian cancer patients [4, 20–23]. Metformin decreases the production of insulin, insulin like growth factor, inflammatory cytokines and vascular endothelial growth factor, and therefore it exerts anti-mitotic, anti-inflammatory and anti-angiogenic effects [23]. Metformin significantly restricts the growth of ovarian cancer cell lines and can potentiate the anti-proliferative effect of cisplatin as both invitro and invivo [24]. Metformin can reduce ovarian cancer risk and may prolong survival among diabetic patients [25]. These findings support that diabetes mellitus can influence the incidence and survival in patients with ovarian cancer [26]. Data from epidemiological reports and meta-analysis support that diabetes increases the risk of colorectal, breast and endometrial cancer, and may associate with poorer survival in colon, pancreas and breast cancer [1]. However, few limited epidemiological studies have investigated the association between diabetes mellitus and ovarian cancer mortality. Their results are inconsistent [27–31]. Our purpose of this study was to describe the impact of comorbid diabetes mellitus on survival in ovarian cancer patients.

## Methods

This retrospective cohort study was approved in accordance with standards of the Institutional Human Subjects Projection Review Board at the Tehran University of Medical Science. Aims of the study were clearly explained for all participants and written informed consent was obtained from all patients prior to beginning the data gathering. Eligible individuals were also assured regarding their confidentiality and anonymity, and they could withdraw at any stage of the study. Eligible subjects were diagnosed with EOC and treated between 2009 and 2016 at Vali-e-Asr Hospital with complete records. We checked fasting blood sugar (FBS) and HbA1C in all of our patients routinely. So, the DM has occurred before the EOC, and there is temporality between exposure and outcome. In this study, we wanted to find that whether there is any difference in PFS and OS between women with diabetes and those without diabetes in this population of EOC patients. Records were reviewed for standard demographic data, presence or absence of diabetes type2, HTN, BMI, pathologic and treatment data (chemotherapy administration approach), PFS (calculated from the time of initiation of chemotherapy until cancer recurrence or progression according to clinical assessment, and rising CA125 or radiographic evidence of recurrence). OS was calculated from initiation of chemotherapy until death or last contact. Those diagnosed with diabetes at the time of preoperative workup were included as diabetes, and patients who developed diabetes after diagnosis of ovarian cancer were included in the other group.

## Data analysis

Descriptive statistics are presented as frequency (percent) for categorical variables and as mean ± (SD) or median and range for continuous variables. Difference between baseline characteristics of patients with and without diabetes compared with chi-squared test for categorical variables and Student’s t test for continuous variables. Univariable differences in OS and PFS between patients with and without diabetes were evaluated using the Kaplan-Meier method and univariate Cox proportional hazard models. Multivariate Cox proportional hazard models were then used to evaluate the relationship between disease status and survival (OS or PFS) after adjustment for age, BMI, parity, stage, grade, histology, debulking status, HTN, menopause status, metformin intake and neoadjuant chemotherapy. STATA version 13 was used for all statistical analyses and likelihood ratio tests for all the hypothesis tests. The proportional hazards assumption for all the Cox regression models was checked using the diagnostic section within STATA.

## Results

Two hundred and fifteen patients who were diagnosed with EOC between 2009 and 2016 met criteria for inclusion. The mean ± (SD) duration of follow-up was 38.77 ± (15.15) months (range 10 to 60 months). The median age of EOC diagnosis was 61 years (range: 44 to 85 years) and median BMI was 26 kg/m^2^ (range 21–32 kg/m^2^). The majority of the patients (71.2%) were undergoing optimal cytoreductive surgery at initial exploration to 1 cm of residual disease. During the 60 months following EOC diagnosis, 95(44.2%) deaths occurred. The OS across all stages for 24, 36, and 60 months were 90.64%(95%CI: 85.70 to 93.93), 69.79%(95%CI: 62.24 to 76.12), and 21.89%(95%CI: 13.47 to 31.63), respectively. Also, the PFS across all stages for 24, 36, and 60 months were 61.46%(95%CI: 48.48 to 73.89), 47.33%(95%CI: 32.42 to 64.77), and 14.66%(95%CI: 8.52 to 27.16), respectively. Of the patients, 37(17.2%) patients had a recorded diagnosis of diabetes. For comparison, we examined the cohort by dividing it into non-diabetic patients (*n* = 178) and diabetic patients (*n* = 37).

**Fig. 1:**
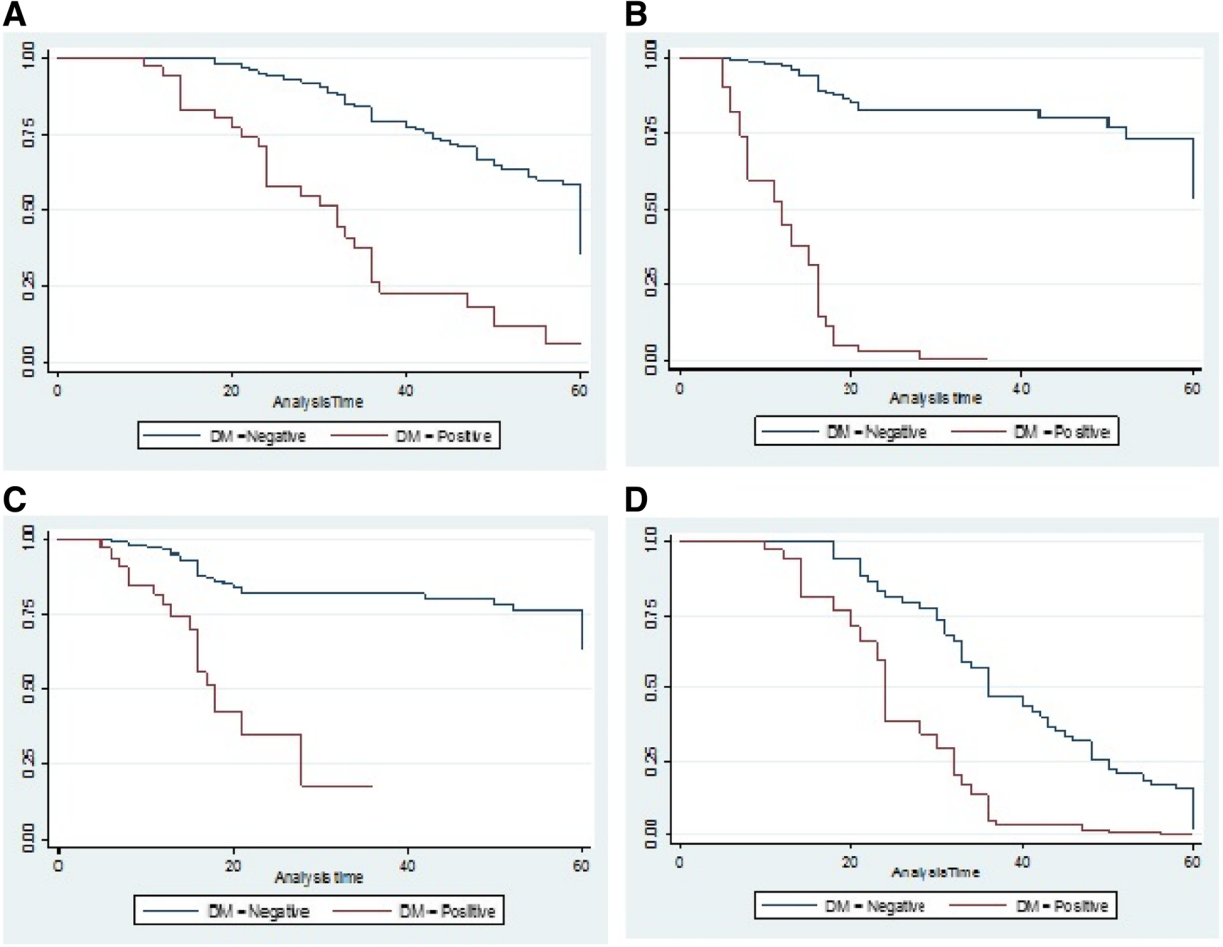
(**a**) Overall survival among patients with versus without diabetes. (**b**) Overall survival among patients with versus without diabetes adjusting for age, BMI, parity, stage, grade, histology, debulking status, HTN, menopause status and neoadjuant chemotherapy. (**c**) Progression-free survival among patients with versus without diabetes. (**d**) Progression-free survival among patients with versus without diabetes adjusting for age, BMI, parity, stage, grade, histology, debulking status, HTN, menopause status and neoadjuant chemotherapy

The mean ± (SD) age of EOC diagnosis of patients with diabetes was 65.35 ± (10.13) which was significantly higher than the mean ± (SD) age of EOC diagnosis of patients without diabetes, 61.30 ± (8.87), (*P* = 0.015) (Table 1). Patients with diabetes had higher BMI (Mean Difference: 0.35, 95%CI: 1.93 to 3.32, *P* < 0.001) and parity (Mean Difference: 0.53, 95%CI: 1.31 to 3.48, P < 0.001), compared to patients without diabetes. Administration of neoadjuant chemotherapy was not different between two groups (10 (5.6%) in non-diabetic and 3 (8.1%) in diabetic patients; *P* = 0.563). Diabetic patients were more likely to have either stage III (81.1%) or stage IV (8.1%) disease than the non-diabetic cohort (*P* = 0.047). Distributions of baseline characteristics for two groups are shown in Table 1. Patients with DM (27.97, 95%CI: 23.63 to 32.30) had a significantly shorter OS rates compared to patients without DM (41.01, 95%CI: 38.84 to 43.17(Fig. 1a). The unadjusted HR for the association between OS time and DM was 4.76 (95%CI: 2.99 to 7.59, *P* < 0.001). Following adjustment for age, BMI, parity, stage, grade, histology, debulking status, HTN, menopause status, metformin intake and neoadjuant chemotherapy the HR was 3.93 (95% CI 2.01 to 7.68; *P* < 0.001) (Fig. 1b). The PFS in patients with DM (14.10, 95%CI: 11.76 to 16.44) was significantly shorter compared to patients without DM (28.83, 95%CI: 26.13 to 31.54(Fig. 1c). The unadjusted HRs for PFS and DM was 5.69 (95% CI:3.05 to 10.61; P < 0.001). After adjustment for age, BMI, parity, stage, grade, histology, debulking status, HTN, menopause status, metformin intake and neoadjuant chemotherapy the HR was 2.73 (95% CI: 1.18 to 6.95; *P* < 0.001) (Fig. 1d).

**Table 1:** Characteristics of the Study Population

	Diabetic Patients (*n* = 37)	Nondiabetic Patients (*n* = 178)	*P*-value
Age at diagnosis (year)^a^	65.35 ± (10.13)	61.30 ± (8.87)	0.015
Follow-up^a^	27.97 ± (13.00)	41.01 ± (14.62)	< 0.001
BMI^a^	28.10 ± (2.25)	25.47 ± (1.87)	< 0.001
Parity^a^	4.45 ± (3.06)	2.05 ± (2.50)	< 0.001
Neoadjuant chemotherapy^b^			
Yes	3(8.1)	10(5.6)	0.563
No	34(91.9)	168(94.4)	
Menopausal status^b^			
Premenopausal	10(27)	83(46.6)	0.029
Postmenopausal	27(73)	95(53.4)	
Stage^b^			
I	1(2.7)	18(10.1)	0.047
II	3(8.1)	42(23.6)	
III	30(81.1)	110(61.8)	
IV	3(8.1)	8(4.5)	
Grade^b^			
1	10(27)	107(60.1)	0.001
2	15(40.5)	38(21.3)	
3	12(32.5)	33(18.5)	
Histology^b^			
Serous	26(70.3)	103(57.9)	0.482
Mucinous	6(16.2)	41(23)	
Endometrioid	4(10.8)	16(9)	
Clear	1(2.7)	17(9.6)	
Transitional	0(0)	1(0.6)	
Debulking status^b^			
Optimal	14(37.8)	139(78.5)	< 0.001
Suboptimal	23(62.2)	37(20.9)	
Unknown	0(0)	1(0.6)	
HTN			
Yes	10(27)	28(15.7)	0.101
No	27(73)	150(84.3)	

^a^: Values given as mean ± SD(standard deviation)

^b^: Values given as number (percentage)

## Discussion

In this retrospective cohort study, we find that DM has negative impact on survival in EOC patients. OS and PFS diabetic group was lower than non-diabetic patients similar to Shah et al. sturdy [2]. Diabetes mellitus is one of the chronic diseases that prevalent with aging and can influence on the incidence and survival in several cancer patients such as ovarian cancer. The association between diabetes and cancer was noted as early as 1914 [32]. Both cancer and diabetes mellitus are multifactorial and have similar risk factor such as age, obesity and life styles. Although the exact mechanism by which diabetes links to cancer is not fully understand, strong association have been demonstrated in pancreatic [33], liver [34], colon [35] and endometrial cancer [36] . Some epidemiological studies investigated the association between DM and survival in the cancer. In a meta-analysis of 17 cohort studies, Lee et al. reported that preexisting diabetes mellitus was associated with increase in cancer-specific mortality and all-cause mortality among prostatic cancer patients [37]. Millis et al. found that colorectal cancer patients with diabetes mellitus has a higher risk for all-cause and cancer-specific mortality [38]. In meta-analysis of 12 cohort studies, Dongyu Zhang et al. reported that diabetes mellitus is associated with a higher all-cause and cancer-specific mortality in ovarian cancer patients [1]. In the meta-analysis of 19 studies, Jung-Yun lee et al. reported that there is a significant association between preexisting diabetes and ovarian cancer incidence [39]. Interestingly, metformin, the first line medication for the treatment of type 2 diabetes, particularly in overweight patients [18, 19] have positive impact on survival in diabetes ovarian cancer patients [4, 20–23]. Metformin decreases the production of insulin, insulin like growth factor, inflammatory cytokines and vascular endothelial growth factor; therefore, it exerts anti-mitotic, anti-inflammatory and anti-angiogenic effects [23]. Metformin significantly restrict the growth of ovarian cancer cell lines and can potentiate the anti-proliferative effect of cisplatin both invitro and invivo [24]. In the meta-analysis of 9 article, Lifeng li et al. reported that metformin can reduce ovarian cancer risk and may prolonged survival among diabetic patients [25]. These findings support that diabetes mellitus can influence on the incidence and survival in patients with ovarian cancer [26]. In addition to hyperglycemia in diabetes, hyperinsulinmia and an increase in bioavailable insulin like growth factor 1 (IGF-1) may play an important role in neoplastic transformation. Changing in the level of the sex hormone and ovarian androgens due to hyperinsulinmia may play a role in ovarian tumor genesis [39]. The lower survival in ovarian cancer patients with diabetes is likely multifactorial. Older age, higher BMI and other medical conditions such as HTN are common in diabetic patients. We attempt to adjust for these variables in our multivariable model. Diabetic patients usually have higher BMI and debulking surgery can be compromised because of higher BMI and comorbid conditions such as HTN during surgery in diabetic patients. In the other hand, physicians maybe more cautions of adverse effect of chemotherapy in diabetic patients and this can effect in adjuvant chemotherapeutic cycle in diabetic patients [8]. In this study, the mean age of diabetic group was significantly higher than non-diabetic, this finding is similar to Bakhru et al. study [26]; although in the study of Shah et al. there wasn’t any difference in the age between two groups. High parity is a conservative factor for EOC development, but in our study, diabetic patients have significantly higher parity, and this can propound the causative effect of diabetes in ovarian cancer. Diabetic patients have higher BMI but this variable couldn’t effect on survival similar to Shah et al. study. This study shows that receiving neoadjuant chemotherapy and suboptimal debulking surgery and having diabetes mellitus can influence on survival in ovarian cancer patients, but after adjusting these variables, diabetes Mellitus had stronger effect on survival with an adjusted HR, 3.93 (95% CI 2.01 to 7.68; *P* < 0.001). The independent prognostic impact of diabetes in ovarian cancer patients may be related to biologic effect of diabetes on cancer cells. Interesting, we found in our study that diabetic patients had more aggressive cancers, with higher grade (P < 0.001), similar to Shah et al. [2] and Bakhru et al. study, and higher stage similar to Bakhru et al. study (*P* = 0.04). Decreased states of tumor differentiation would be consistent with an increase in tumor “stemness” [26]. Several limitations exist within this study, first there may be several medical condition, different habitus and life style and different degree of physical activity that can act as confounding factors and influence on survival. Second, we hadn’t data about degree of diabetic control, type of diabetes and duration of having diabetes in ovarian cancer patients. We considered patients to have diabetes, only if they had a recorded diagnosis or and anti-hyperglycemic medication was listed. We didn’t routinely check fasting blood sugar (FBS) and HbA_1_C in all of our patients; given that up to 27% of diabetes can be undiagnosed [2]. So, the association between DM, OS and PFS in patients with EOC should be considered with caution due to the small number of included participants, misclassification bias based on diagnostic method of DM and the residual confounding regarding unmeasured variables such as life style habits, physical activity and medical condition. The relation between ovarian cancer and diabetes is complex. We suggest doing prospective cohort studies in ovarian cancer patients to evaluate the effect of diabetes mellitus and its treatment modalities, on ovarian cancer patient’s survival.

## Conclusions

DM can negatively effect on PFS and OS in EOC patients independent of the effect of other variables.

## Abbreviations

BMI: Body mass index
DM: Diabetes mellitus
EOC: Epithelial ovarian cancer
HR: Hazard ratio
HTN: Hypertension
IGF-1: Insulin growth factor-1
OS: Overall survival
PFS: Progression free survival
